# Specific microRNAs for Heart Failure: Reference Values in Whole Blood

**DOI:** 10.3390/biomedicines13102559

**Published:** 2025-10-20

**Authors:** Radka Sigutova, Lukas Evin, Pavlina Kusnierova, David Stejskal, Frantisek Vsiansky, Eva Bace, Eliska Kufova, Gabriela Kubikova, Zdenek Svagera, Marian Branny, Jan Vaclavik

**Affiliations:** 1 Institute of Laboratory Medicine, Department of Clinical Biochemistry, University Hospital Ostrava, 708 52 Ostrava, Czech Republic; pavlina.kusnierova@fno.cz (P.K.); david.stejskal@fno.cz (D.S.); frantisek.vsiansky@fno.cz (F.V.); gabriela.kubikova@fno.cz (G.K.); zdenek.svagera@fno.cz (Z.S.); 2 Institute of Laboratory Medicine, Faculty of Medicine, University of Ostrava, 700 30 Ostrava, Czech Republic; 3 Department of Epidemiology and Public Health, Faculty of Medicine, University of Ostrava, 700 30 Ostrava, Czech Republic; 4 Department of Internal Medicine and Cardiology, University Hospital Ostrava, 708 52 Ostrava, Czech Republic; lukas.evin@fno.cz (L.E.); eliska.kufova@fno.cz (E.K.); marian.branny@fno.cz (M.B.); jan.vaclavik@fno.cz (J.V.); 5 Research Center for Internal and Cardiovascular Diseases, Faculty of Medicine, University of Ostrava, 700 30 Ostrava, Czech Republic; 6 BioVendor—Laboratory Medicine Inc., 621 00 Brno, Czech Republic; bace@biovendor.com

**Keywords:** miRNA (microRNA), heart failure, reference values, microRNA enzymatic immunoassay (miREIA) method

## Abstract

**Background:** The objective of this study was to validate reference values for eight selected microRNAs (miRNAs) in a population of healthy individuals. The selected miRNAs (hsa-miR-21-5p, hsa-miR-23a-3p, hsa-miR-142-5p and hsa-miR-126-3p, hsa-miR-499a-5p, hsa-miR-195-5p, hsa-miR-1-3p, hsa-miR-29a-3p) have an important role in heart failure. **Methods:** Ninety-nine individuals were selected for this study. Specific microRNAs were isolated from whole blood and quantified using the microRNA enzymatic immunoassay (miREIA) method. Reference intervals were evaluated with respect to age and sex. Statistical analyses were performed using MedCalc (v22.021) and R software, Version 4.1.2. **Results:** Reference values (2.5th and 97.5th percentile values and their 90% confidence intervals) were determined for hsa-miR-21-5p: 1.45 to 96.3 pmol/L, hsa-miR-23a-3p: 13.0 to 432 pmol/L, hsa-miR-126-3p: 5.67 to 66.5 pmol/L, hsa-miR-142-5p: 37.4 to 293 pmol/L, hsa-miR-195-5p: 11.5 to 254 pmol/L, hsa-miR-1-3p: 50.6 to 1800 pmol/L, hsa-miR-499a-5p: 8.90 to 82.5 pmol/L and hsa-miR-29a-3p: 22.9 to 210 pmol/L. The median age of the included individuals was 44 years (range: 23–75 years). No sex-related differences were observed in the reference intervals of the microRNAs (*p* < 0.05). Except for hsa-miR-21-5p (RS = −0.208; *p* = 0.043), no significant age-related associations were found for the other microRNAs (*p* < 0.05). However, due to the limited number of individuals in the stratified subgroups, reference intervals were not calculated for these subgroups. **Conclusions:** In this study, reference intervals for eight specific miRNAs associated with heart failure were determined. The results are unique for assessment in further clinical research, given that reference intervals in absolute values have not yet been published.

## 1. Introduction

Circulating microRNAs are small non-coding RNA molecules, 19–24 nucleotides in length, that significantly influence physiological and pathophysiological cellular processes [1]. It is well established that these factors play a pivotal role in regulating cellular functions within the cardiovascular system, including cardiomyocytes, fibroblasts, smooth muscle cells, and endothelial cells [2,3]. At the molecular level, they mediate post-transcriptional regulation of genes involved in physiological processes such as proliferation, differentiation, and apoptosis. Dysregulation of these factors can contribute to severe pathological conditions. Cardiovascular diseases, particularly heart failure, remain leading causes of morbidity and mortality worldwide, especially in developed countries. Despite advances in diagnostic precision, approximately 50% of patients diagnosed with heart failure die within 5 years [4,5]. Circulating miRNAs, detectable extracellularly in the bloodstream, have emerged as potential biomarkers of heart failure [3].

The aim of this study was to establish reference values for selected specific miRNAs (*hsa-miR-21-5p*, *hsa-miR-23a-3p*, *hsa-miR-142-5p*, *hsa-miR-126-3p*, *hsa-miR-499a-5p*, *hsa-miR-195-5p*, *hsa-miR-1-3p*, and *hsa-miR-29a-3p*) associated with pathological changes leading to cardiovascular disease, including heart failure diagnosis [6]. These miRNAs were selected on the basis of literature searches of scientific publications. The publicly available miRBase repository (http://mirbase.org/, accessed on 12 March 2018) is a valuable resource used to search for existing miRNA sequences and their annotations [7]. It provides comprehensive data on miRNA sequences and their predicted targets. This information is available for download [8]. The miREIA method was used to establish reference ranges for miRNAs, as it enables absolute quantification in pmol/L. This aspect is essential for the future development and clinical implementation of miRNA-based diagnostic tools. It allows results to be interpreted in relation to population-based reference values, in a manner analogous to established biochemical markers.

## 2. Materials and Methods

### 2.1. Study Population

The study included 99 healthy adults aged 23–75 years. Six individuals were excluded due to extreme values (outliers) or insufficient data quality. All participants provided written informed consent. We performed transthoracic echocardiography at the Department of Internal Medicine and Cardiology, University Hospital Ostrava, before collecting blood samples. We collected whole blood samples at the Department of Clinical Biochemistry and Hematology, Institute of Laboratory Medicine, University Hospital Ostrava, to analyze classical biochemical and hematological markers (Table 1).

Whole blood samples were collected in two tubes from participants who met predefined inclusion and exclusion criteria. Inclusion criteria were availability of clinical and laboratory data and absence of current pharmacological treatment. Exclusion criteria included severe liver or kidney dysfunction based on laboratory results, history or clinical signs of cardiovascular disease, diabetes mellitus, malignancy, or any acute or chronic inflammatory condition within the last 6 months. Blood samples were collected in the morning from selected individuals who were fasting and had not engaged in significant physical activity on the day of sampling. Samples from healthy individuals were collected and processed pseudonymously using predetermined identification numbers.

### 2.2. Samples

For the analysis of classical biochemical parameters (glucose, HbA1c, urea, creatinine, AST, ALT, CRP, NT-proBNP; Table 1) and haematological blood count parameters (HCT, Hb, PLT, WBC; Table 1), blood was collected using Sarstedt tubes (S-Monovette^®^ K3 EDTA, 2.6 mL, red cap; S-Monovette^®^ Lithium heparin gel LH, 4.9 mL, orange cap; S-Monovette^®^ Serum Gel CAT, 4.9 mL, brown cap; Sarstedt AG & Co., KG, Nümbrecht, Germany). For the determination of specific microRNAs, 2.5 mL of whole blood was drawn into two PAXgene^®^ Blood RNA tubes (Vacutainer^®^ PAXgene, PreAnalytiX GmbH, a Qiagen/BD Company, Hombrechtikon, Switzerland). Whole blood samples were stored upright at room temperature for 2 h, then frozen at −20 °C for 24 h, and subsequently stored at −80 °C until analysis (isolation).

### 2.3. Determination of Classical Biomarkers

Biochemical markers (glucose, urea, creatinine, AST, ALT, CRP, and NT-proBNP; Table 1) were measured in serum or plasma after centrifugation (at 2500× *g*, 4 °C, for 6 min) at the Institute of Laboratory Medicine using the AU5800 analyzer (Beckman Coulter, Inc., Brea, CA, USA) or ADVIA Centaur^®^ XPT (Siemens Healthcare Diagnostics Inc., Tarrytown, NY, USA). Glycated hemoglobin was quantified from whole blood using the Tosoh G8 analyzer (model HLC-723G8; Tosoh Bioscience, Inc., South San Francisco, CA, USA). Hematological parameters (Table 1) were analyzed with the Sysmex XN-9000 analyzer (Sysmex Corporation, Kobe, Hyogo, Japan). Biomarker assessment was performed immediately upon sample arrival at the laboratory.

### 2.4. Isolation of miRNA

Isolation of specific miRNAs was performed according to the manufacturer’s instructions (BioVendor—Laboratory Medicine Corp., Brno, Czech Republic) using the miRNA isolation kit (cat. no. RIK001). PAXgene tubes were incubated at room temperature for 2 h, then mixed and centrifuged at 4500× *g* and 18 °C for 12 min. The supernatant was discarded, the remaining sample was mixed, and 4 mL of RNAse-free molecular biology-grade water (cat. no. 95284, Sigma-Aldrich^®^, Burlington, MA, USA) was added. Samples were centrifuged again under the same conditions, the supernatant removed, and the pellet thoroughly homogenized by mixing. The homogenate was transferred into 2 mL microtubes, and 800 µL of Qiazol lysis buffer (cat. no. 79306, Qiagen, Venlo, The Netherlands) was added to each. Samples were mixed and incubated at room temperature for 5 min. After 160 µL of chloroform was added to each tube, samples were mixed and incubated for an additional 5 min at room temperature, followed by centrifugation at 12,000× *g* for 20 min. The upper aqueous phase was transferred to new 2 mL microtubes, and 5 µL of diluted “SPIKE-in Control” (exogenous *cel-miR-39-3p*, 10 pmol/L) and an equal volume of buffer (1:1) were added.

A defined amount of synthetic *cel-miR-39-3p* was added to each sample during the isolation step to serve as an internal control for miRNA recovery. This synthetic miRNA was quantified in parallel with target miRNAs, and the resulting values were used to calculate an isolation efficiency coefficient, which was applied to normalize the measured concentrations of specific miRNAs.

Each sample was washed in five steps using centrifugal filter microcolumns inserted into collection tubes. Following centrifugation (30 s at 8000× *g*), the eluate was removed. The column was then transferred to a new microtube, and 50 µL of RNAse-free water was added. After incubation for 1 min, samples were centrifuged (1 min at 11,000× *g*). The final RNA isolate was aliquoted into labelled microtubes and stored at −80 °C until analysis.

### 2.5. Determination of miRNA

Concentrations of specific miRNAs were measured using microRNA enzyme immunoassay kits (miREIA, BioVendor). The following kits were used for the determination: *hsa-miR-21-5p* miREIA (cat. no. RDM0001H), *hsa-miR-23a-3p* miREIA (cat. no. RDM0009H), *hsa-miR-142-5p* miREIA (cat. no. RDM0013H), *hsa-miR-126-3p* miREIA (cat. no. RDM0018H), *hsa-miR-499a-5p* miREIA (cat. no. RDM0016H), *hsa-miR-1-3p* miREIA (cat. no. RDM0019H), *hsa-miR-29a-3p* miREIA (cat. no. RDM0034H), *hsa-miR-195-5p* miREIA (cat. no. RDM0036H), and a kit for the determination of exogenous control *cel-miR-39-3p* (cat. no. RDM0000C).

Determination was performed following the manufacturer’s instructions. MiRNA isolates were appropriately diluted, and specific miRNAs were hybridized with biotin-labelled complementary DNA probes (specific DNA oligonucleotides). Hybridization was carried out using a Turbo Cycler Lite TCLT-9620 (Blue-Ray Biotech, New Taipei City, Taiwan, China). The assay was conducted in microtiter wells coated with a monoclonal antibody specific for DNA/miRNA heterohybrids. After incubation and washing, streptavidin–horseradish peroxidase (HRP) conjugate was added, followed by further incubation and washing steps. The chromogenic substrate tetramethylbenzidine (TMB) was then applied, and after incubation, the reaction was stopped by adding acidic STOP solution. Absorbance of the resulting yellow product, proportional to miRNA concentration, was measured at 450 nm using a DSX Automated Microplate ELISA system (model 1DXC2087, DYNEX Technologies, Chantilly, VA, USA).

Data were technically normalized by multiplying each miRNA concentration by the isolation efficiency coefficient. This coefficient was calculated as the ratio between the expected concentration of the exogenous control *cel-miR-39-3p* added prior to isolation and the concentration measured in the samples by *cel-miR-39-3p* miREIA.

### 2.6. Statistical Analysis

Reference intervals for specific miRNAs were calculated using the Robust method recommended for small sample sizes in MedCalc (v22.021) [9,10]. Graphical data analysis was performed in R software [11]. Potential outliers were assessed using Tukey’s method. Data distribution was evaluated with the Shapiro-Wilk test. Due to non-normal distribution, differences between two independent groups (men, *n* = 36; women, *n* = 63) were analyzed with the non-parametric Wilcoxon test. Associations between specific miRNA levels and age or body mass index (BMI) were assessed by Spearman’s rank correlation (R_s_). Statistical significance was set at *p*  <  0.05.

## 3. Results

Values of classical biochemical and hematological markers characterizing the selected study population are presented in Table 1.

**Figure 1:**
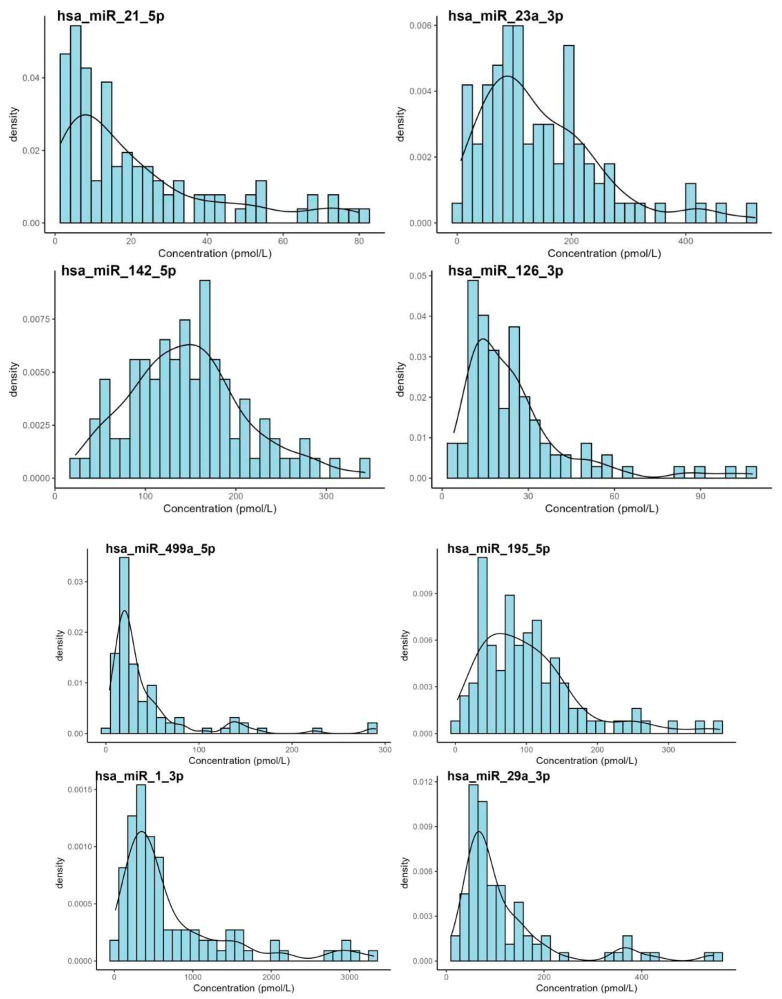
Distribution of the miRNA values using histograms. The distribution profiles of most miRNA concentrations measured in whole blood samples from healthy individuals demonstrated right-skewed, non-Gaussian patterns with variable degrees of dispersion. Notably, certain miRNAs, such as *hsa-miR-1-3p*, exhibited a broad concentration range, indicative of substantial biological variability. These findings suggest that non-parametric statistical methods may be more suitable for subsequent data analyses.

Basic descriptive statistics and Shapiro-Wilk normality test results indicated a non-normal distribution of the data (*p* < 0.001; Table 2, Figure 1).

**Figure 2:**
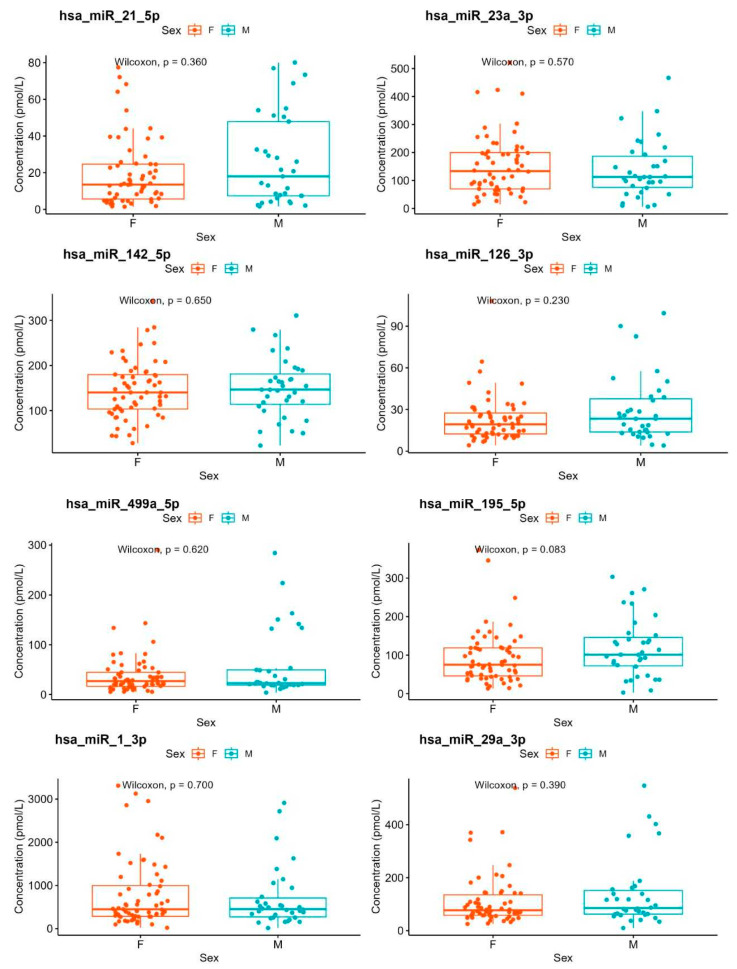
Box-plot graphs showing the lack of dependence of miRNA values on sex (F = female, M = male).

No statistically significant differences in reference values were observed between sexes (Table 3; Figure 2) or across BMI categories (*p* ≥ 0.05; Table 3).

**Figure 3:**
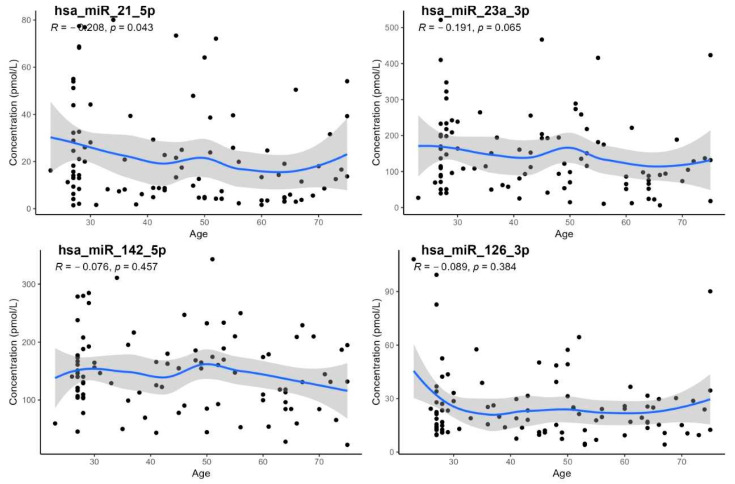

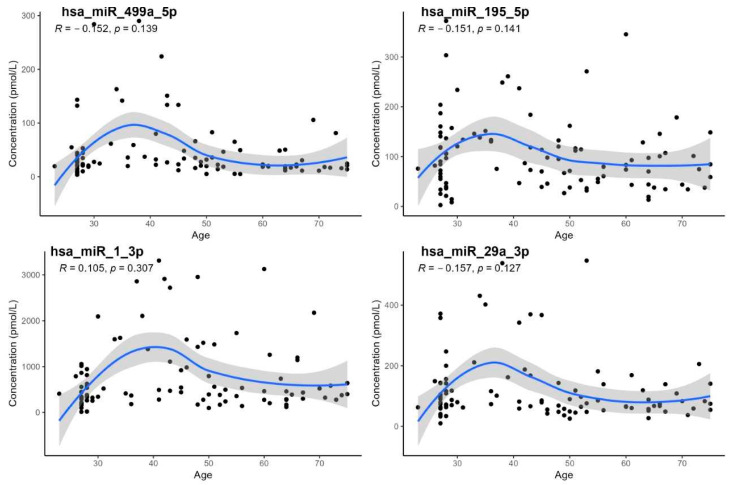
The relationships between specific miRNA concentrations and age were assessed using nonlinear regression models. While most miRNAs showed no statistically significant correlation with age, *hsa-miR-21-5p* demonstrated a modest but significant negative association (R = −0.208, *p* = 0.043). The observed trends indicate age-related changes in the expression levels of certain miRNAs, although these patterns were generally weak or nonsignificant for the majority of the miRNAs analyzed. Confidence intervals around the regression curves illustrate the variability of the data across the studied age range.

Except for *hsa-miR-21-5p*, no statistically significant association with age was observed (*p* < 0.05; Table 3, Figure 3). Although *hsa-miR-21-5p* showed a weak but significant negative correlation with age (R_S_ = −0.208; *p* = 0.043), reference intervals were not stratified by age due to the limited number of individuals in each subgroup.

Spearman’s rank correlation of data with age generally had a slightly decreasing character (Figure 3).

Reference intervals for miRNAs (excluding outliers) are presented in Table 4 for both sexes and the entire age range of healthy individuals.

## 4. Discussion

This study presents the first set of reference intervals for selected specific miRNAs in whole blood, determined using the miREIA method, which enables the quantification of absolute concentrations in pmol/L units. The miREIA method offers several key advantages: in addition to providing high accuracy and robust measurement, it eliminates the need for enzymatic amplification, thereby significantly reducing the risk of technical variability. As a result, it is capable of providing reproducible results with minimal influence from both preanalytical and analytical factors that may affect other methods. RT-qPCR remains the gold standard for miRNA quantification, particularly due to its sensitivity and wide availability; however, its results are susceptible to variability introduced during the reverse transcription and amplification steps. The strong concordance between miREIA and RT-qPCR data (Pearson correlation coefficient > 0.9) confirms that miREIA is a valid and promising alternative, offering improved standardization and reliability for clinical applications [12,13].

Cardiovascular diseases remain one of the leading causes of morbidity and mortality worldwide [14], and miRNAs are a promising tool for improving the diagnosis and prognosis of heart diseases. Compared with traditional biomarkers such as NT-proBNP, miRNAs are stable in circulation and, importantly, reflect specific pathophysiological processes such as fibrosis or hypertrophy [15,16].

A major clinical and research benefit of this study may lie in the more accurate and standardized assessment of miRNAs, which could enhance both the diagnosis and prognosis of cardiovascular diseases. These reference values may serve as a foundation for the development of novel diagnostic algorithms combining multiple biomarkers and supporting personalized medicine.

The main findings of this study include:

- The establishment of reference intervals for selected miRNAs (hsa-miR-1-3p, hsa-miR-21-5p, hsa-miR-23a-3p, hsa-miR-29a, hsa-miR-126-3p, hsa-miR-142-3p, hsa-miR-195-5p, hsa-miR-499a-5p) in a healthy population,
- The demonstration of a statistically significant age dependency only for hsa-miR-21-5p; however, age-specific reference intervals could not be calculated due to the low number of subjects in individual age groups,
- The lack of confirmation of age dependency for hsa-miR-29a, despite some earlier studies having reported such an association [17].

Our results suggest that *hsa-miR-21-5p* is sensitive to age-related changes, which is consistent with previous findings on its role in fibrotic remodeling and the activation of signaling pathways in cardiac fibroblasts [17,18,19]. In contrast, for *hsa-miR-29a*, which has been associated in some studies with hypertrophy and fibrotic processes [20,21], we found no significant correlation with age in our cohort.

This study did not include groups of patients with cardiovascular diseases, as the aim was to determine reference intervals in a healthy population, which is the standard approach for defining normal values. A comparison of these reference values with data from patients with disease is planned for future research and will be essential in evaluating the clinical relevance of miRNAs as biomarkers.

Reference intervals provide an important starting point for further research and clinical applications, particularly in the evaluation of deviations in miRNA levels in patients with cardiovascular diseases. For example, *hsa-miR-1-3p* and *hsa-miR-21-5p* have been identified as potential diagnostic markers in patients with HFrEF (heart failure with reduced ejection fraction), showing high predictive value (area under the receiving operator characteristic curve [AUC] = 0.888 and 0.707, respectively) [15]. Other studies have shown that *hsa-miR-1-3p* is associated with left ventricular function in hypertrophic cardiomyopathy (HCM) and can differentiate dilated cardiomyopathy (DCM) from HCM [22].

Levels of *hsa-miR-23a-3p* were elevated in patients with both types of cardiomyopathy [20], and together with *hsa-miR-21-5p* levels, were identified as significant predictors of hospitalization for heart failure and cardiovascular death [23].

*Hsa-miR-126-3p* and *hsa-miR-142-5p* demonstrated very high AUC values when distinguishing healthy children from patients with DCM [24], and their potential has also been confirmed in the adult population [25,26,27]. *Hsa-miR-499a-5p* also shows high diagnostic value, especially in patients with HCM caused by MYH7 mutation (AUC = 0.95; sensitivity 0.86; specificity 0.91) [28].

While *hsa-miR-195-5p* is associated with hypertrophy and arrhythmias primarily at the cellular and animal levels [29,30], its potential use as a biomarker in humans remains open for future research.

When interpreting the established reference intervals, several biological and regulatory aspects of the selected miRNAs must be considered. Some of the miRNAs mentioned have extracardiac expression, which may reduce their specificity for cardiac pathologies. *Hsa-miR-21-5p* and *hsa-miR-142-5p* are also expressed in macrophages, immune cells, and fibroblasts [19,31], whereas *hsa-miR-126-3p* is endothelial-specific [32]. In contrast, *hsa-miR-1-3p* and *hsa-miR-499a-5p* are considered heart-specific miRNAs [33].

Application of the reference intervals established in this study requires strict adherence to preanalytical conditions and standardization of sample collection timing.

Some of the aforementioned miRNAs may be candidates for circadian variability, which is relevant in terms of the potential influence of sample collection time during the day. According to Schober et al., *hsa-miR-21* levels in macrophages vary depending on the time of day, with lower levels observed in the morning, coinciding with increased cell death. This condition may contribute to the progression of atherosclerosis [34].

The expression of *hsa-miR-142-3p* is directly regulated by the CLOCK/BMAL1 complex, while *hsa-miR-142-3p* itself suppresses the expression of the *Bmal1* gene. This indicates the existence of a feedback regulatory loop between this miRNA and key circadian rhythm genes [35]. *Hsa-MiR-29a/b/c* reduce the expression of the *hPER1* gene, which is essential for proper circadian clock function [36].

According to the study by Schneider et al., *hsa-miR-21* and *hsa-miR-126* are more suitable for longitudinal monitoring of changes depending on the (de)compensated state of patients with heart failure and are associated with all-cause mortality [37].

Cardiac-specific miRNAs, such as *hsa-miR-499a-5p* and *hsa-miR-1-3p*, are suitable as acute diagnostic markers of myocardial infarction, as well as for identifying patients at increased risk of death during the first year after acute myocardial infarction, especially when combined with NT-proBNP levels [38].

For long-term monitoring of fibrosis, hypertrophy, and structural changes after infarction or in cardiomyopathy, *hsa-miR-29a-3p* appears to be suitable [39].

In this study, preanalytical influences were minimized through a standardized sample collection procedure. Samples were collected in the morning, in a fasting state, and in accordance with recommended methodological guidelines. To further enhance the clinical relevance of the established reference intervals, future studies should evaluate the intra-individual stability of the selected miRNA levels over a longer time period and consider the potential impact of additional physiological factors. Longitudinal monitoring could help determine whether the selected miRNAs are more suitable for diagnostic purposes or for tracking disease progression.

### Limitation

The first possible limitation of this study is the uneven number of individuals in the groups by sex (men, *n* = 36; women, *n* = 63). Second, although in the case of *hsa-miR-21-5p* the dependence of values on age was demonstrated, due to the small representation of individuals in the distribution groups, the reference intervals were not calculated further.

## 5. Conclusions

The established reference intervals provide an important tool for more accurate clinical assessment. This work presents the first set of reference intervals for selected miRNAs relevant to cardiovascular biology (*hsa-miR-1-3p*, *hsa-miR-21-5p*, *hsa-miR-23a-3p*, *hsa-miR-29a-3p*, *hsa-miR-126-3p*, *hsa-miR-142-5p*, *hsa-miR-195-5p*, and *hsa-miR-499a-5p*) measured in whole blood using the immunochemical miREIA method, which enables accurate and reproducible quantification in absolute units. The results represent an important starting point for the interpretation of these miRNA levels in a clinical context and may contribute to their more effective use as biomarkers in the diagnosis and monitoring of cardiovascular diseases.

Future research should assess the temporal stability of these levels, confirm their circadian dynamics, and further develop the use of miRNAs within integrated diagnostic algorithms aimed at personalized medicine.

## Figures and Tables

**Table 1:** Basic biochemical markers characterizing healthy individuals (*n* = 99) included in the reference interval study for miRNA determination.

	Mean	Median	SD	Minimum	Maximum	Normality Test (*p*-Value)
Age	44.9	44.0	15.8	23.0	75.0	<0.001
BMI	25.6	25.0	4.80	16.7	40.9	<0.001
Leu [10^9^/L]	6.36	6.06	1.66	4.10	8.91	<0.001
Hb [g/L]	139	139	12	120	169	0.594
Hct [%]	46.1	40.8	4.19	35.1	41.7	<0.001
Urea [mmol/L]	4.78	4.80	1.26	1.90	8.00	0.017
Cr [µmol/L]	74.5	72.0	17.0	54.0	105	0.022
ALT [µkat/L]	0.436	0.330	0.298	0.130	0.710	<0.001
AST [µkat/L]	0.437	0.410	0.158	0.230	0.740	<0.001
NT-proBNP [ng/L]	68.2	46.0	45.9	35.0	252	<0.001
FPG [mmol/L]	5.03	5.09	0.382	4.00	5.60	<0.001
HbA1c [mmol/mol]	35.3	35.5	4.37	29.0	41.0	<0.001
CRP [mg/L]	2.20	1.30	2.50	0.200	7.90	<0.001

Note: Data (*n*, number of individuals) are presented as mean, median, standard deviation (SD), minimum and maximum values of the set. *p*-Values are for the Shapiro-Wilk normality test significance is indicated at alpha = 0.05. Abbreviations: Leu, leukocyte; Hb, hemoglobin; Hct, hematocrit; Cr, creatinine; ALT, alanine aminotransferase; AST, aspartate aminotransferase; NT-proBNP, N-terminal pro-B-type natriuretic peptide; FPG, fasting plasma glucose; HbA1c, glycated hemoglobin A1c; CRP, C-reactive protein.

**Table 2:** Shapiro–Wilk normality test of measured concentrations (*n* = 99) of specific miRNAs (pmol/L), including mean, median, standard deviation (SD), minimum and maximum values, and corresponding *p*-values (*p* < 0.05).

miRNA	Mean	Median	SD	Min.	Max.	Shapiro-Wilk (*p*-Value)
*hsa-miR-21-5p*	25.2	14.3	26.9	1.46	128	<0.001
*hsa-miR-23a-3p*	156	119	132	6.31	920	<0.001
*hsa-miR-142-5p*	155	145	114	21.1	1080	<0.001
*hsa-miR-126-3p*	28.0	21.3	26.2	4.05	156	<0.001
*hsa-miR-499a-5p*	54.8	24.7	82.3	3.79	511	<0.001
*hsa-miR-195-5p*	106	88.6	79.4	2.68	454	<0.001
*hsa-miR-1-3p*	862	468	1090	13.4	8740	<0.001
*hsa-miR-29a-3p*	136	82.3	163	9.81	1250	<0.001

**Table 3:** Evaluation of miRNA levels (*n* = 99) according to sex (male, female), age range (23–75 years), and BMI.

	Wilcoxon Test, Sex	Correlation on Age		Correlation on BMI	
miRNA	*p*-Value	R_S_	*p*-Value	R_S_	*p*-Value
*hsa-miR-21-5p*	0.360	−0.208	0.043	−0.057	0.590
*hsa-miR-23a-3p*	0.570	−0.191	0.065	0.005	0.960
*hsa-miR-126-3p*	0.230	−0.089	0.384	−0.086	0.400
*hsa-miR-142-5p*	0.650	−0.076	0.457	0.016	0.880
*hsa-miR-195-5p*	0.083	−0.151	0.141	−0.140	0.160
*hsa-miR-1-3p*	0.700	0.105	0.307	0.100	0.310
*hsa-miR-499a-5p*	0.620	−0.152	0.139	−0.180	0.160
*hsa-miR-29a-3p*	0.390	−0.157	0.127	−0.140	0.180

Note: The association between miRNA levels and sex was assessed using the Wilcoxon rank-sum test for two independent groups (*p* < 0.05). Correlations between miRNA levels and age or BMI were evaluated by Spearman’s rank correlation coefficient (R_S_; *p* < 0.05). Abbreviations: R_S_, Spearman’s rank correlation coefficient; BMI, body mass index.

**Table 4:** Reference intervals (2.5th–97.5th percentiles, with 90% confidence intervals) for specific miRNAs (pmol/L) in both sexes (female and male) across the age range of 23–75 years.

miRNA	*n*	Outliers	2.5th	90% Confidence Interval	97.5th	90% Confidence Interval
*hsa-miR-21-5p*	95	4	1.45	1.04–2.04	96.3	78.9–120
*hsa-miR-23a-3p*	94	5	13.0	7.78–21.2	432	370–496
*hsa-miR-126-3p*	95	4	5.67	4.70–6.84	66.5	55.2–72.7
*hsa-miR-142-5p*	97	2	37.4	26.9–49.9	293	269–318
*hsa-miR-195-5p*	95	4	11.5	7.06–17.5	254	222–288
*hsa-miR-1-3p*	88	11	50.6	34.4–75.5	1800	1410–2250
*hsa-miR-499a-5p*	86	13	8.90	4.71–7.35	82.5	67.1–98.8
*hsa-miR-29a-3p*	87	12	22.9	18.5–28.4	210	177–243

## Data Availability

The data that support the findings of this study are available on request from the corresponding author. The data are not publicly available due to privacy or ethical restrictions.

## Abbreviations

The following abbreviations are used in this manuscript:

ALT	alanine aminotransferase
AST	aspartate aminotransferase
AUC	area under the receiver operating characteristic curve
BMI	body mass index
CRP	C-reactive protein
DCM	dilated cardiomyopathy
ERK-MAP kinase	extracellular signal-regulated kinase–mitogen-activated protein kinase
FPG	fasting plasma glucose
Hb	hemoglobin
HbA1c	glycated hemoglobin A1c
HCM	hypertrophic cardiomyopathy
Hct	hematocrit
HRP	horseradish peroxidase
HFrEF	heart failure with reduced ejection fraction
IL-6	interleukin 6
Leu	leukocyte
LVEF	left ventricular ejection fraction
miRNA	microRNA
miREIA	microRNA enzymatic immunoassay
MYH7	gene encoding β-myosin heavy chain
NT-proBNP	N-terminal pro-B-type natriuretic peptide
PLT	platelet
RS	Spearman’s rank correlation coefficient
ROC	receiver operating characteristic
RT-qPCR	reverse transcription quantitative polymerase chain reaction
TMB	tetramethylbenzidine (chromogenic substrate)
WBC	white blood cell
